# Cooperation of DNA-PKcs and WRN helicase in the maintenance of telomeric D-loops

**DOI:** 10.18632/aging.100141

**Published:** 2010-04-30

**Authors:** Rika Kusumoto-Matsuo, Patricia L. Opresko, Dale Ramsden, Hidetoshi Tahara, Vilhelm A. Bohr

**Affiliations:** ^1^ Laboratory of Molecular Gerontology, National Institute on Aging, National Institutes of Health, Baltimore, MD 21224, USA; ^2^ Department of Environmental and Occupational Health, University of Pittsburgh Graduate School of Public Health, Pittsburgh, PA 15219, USA; ^3^ Department of Biochemistry and Biophysics, University of North Carolina at Chapel Hill, Chapel Hill, NC 27599, USA; ^4^ Curriculum in Genetics and Molecular Biology, University of North Carolina at Chapel Hill, Chapel Hill,; NC 27599, USA; ^5^ Lineberger Comprehensive Cancer Center, University of North Carolina at Chapel Hill, Chapel Hill, NC 27599, USA; ^6^ Department of Cellular and Molecular Biology, Division of Integrated Medical Science, Graduate School of Biomedical Sciences, Hiroshima University, Hiroshima 734-8551, Japan; ^7^ Present address: Pathogen Genomics Center, National Institute of Infectious Diseases, 4-7-1 Gakuen, Musashi-murayama, Tokyo 208-0011, Japan

**Keywords:** RecQ, WRN, DNA-PKcs, telomere, aging, DNA repair

## Abstract

Werner syndrome is an inherited human progeriod syndrome caused by mutations in the gene encoding the Werner Syndrome protein, WRN.  It has both 3'-5' DNA helicase and exonuclease activities, and is suggested to have roles in many aspects of DNA metabolism, including DNA repair and telomere maintenance. The DNA-PK complex also functions in both DNA double strand break repair and telomere maintenance.  Interaction between WRN and the DNA-PK complex has been reported in DNA double strand break repair, but their possible cooperation at telomeres has not been reported.  This study analyzes thein vitro and in vivo interaction at the telomere between WRN and DNA-PKcs, the catalytic subunit of DNA-PK.  The results show that DNA-PKcs selectively stimulates WRN helicase but not WRN exonuclease in vitro, affecting that WRN helicase unwinds and promotes the release of the full-length invading strand of a telomere D-loop model substrate.  In addition, the length of telomeric G-tails decreases in DNA-PKcs knockdown cells, and this phenotype is reversed by overexpression of WRN helicase.  These results suggest that WRN and DNA-PKcs may cooperatively prevent G-tail shortening in vivo.

## Introduction

Werner syndrome (WS) is a hereditary disorder associated with symptoms of premature aging, including early onset of cataracts, osteoporosis, atherosclerosis and cancer [1,2].  The cellular phenotype of WS includes premature cellular senescence, telomere dysfunction and chromosome instability.  WS is caused by mutations in the gene encoding the Werner syndrome protein (WRN), a multifunction protein that possesses 3'-5' DNA helicase, 3'-5' DNA exonuclease, branch migration, and strand annealing activities [3-8].  WRN helicase is active on a wide variety of DNA substrates, with preference for forked duplex molecules and structures at telomeric DNA [9].

 Telomeres are nucleoprotein structures at the ends ofeukaryotic chromosomes. In humans, telomeric DNA includes a duplex region containing tandem repeats of the sequence 5'-TTAGGG-3' and telomeric 3'-G-overhang, so called G-tail.  The telomere DNA loops back on itself forming a lariat t-loop structure, where the G-tail invades the duplex telomeric repeats and forms a D loop (displacement loop) that stabilizes the t-loop [10].  A complex of six human telomere binding proteins, called shelterin, has been identified [11].  These include TRF1, TRF2, TIN2, RAP1, TPP1 and POT1.  Shelterin promotes formation of a t-loop, which is critical for protecting the G-tail and maintaining telomere length and structure.  WRN has also been detected in telomere complexes.  It interacts with TRF2 and POT1, and regulates telomere processing during S phase [12-14].  This WRN function is biologically important, because WS fibroblasts display accelerated telomere erosion and stochastic telomere loss [15], and WS lymphoblasts show erratic telomere length dynamics [15-17].  The DNA-PK complex, which is composed of a catalytic subunit, DNA-PKcs, regulatory subunits Ku70, and Ku80, is a DNA damage sensing serine-threonine protein kinase that is critical for repair of DNA double strand breaks.  This complex was found at telomeres and DNA-PKcs-deficient cells also exhibit dysfunctional telomeres [18,19].  In addition to the similar defects of telomere in WS cells and DNA-PKcs deficient cells, DNA-PKcs interacts with and phosphorylates WRN in response to DNA double-strand breaks [20-22].  Thus, these two proteins may also cooperate in telomere metabolism.

**Figure 1.**
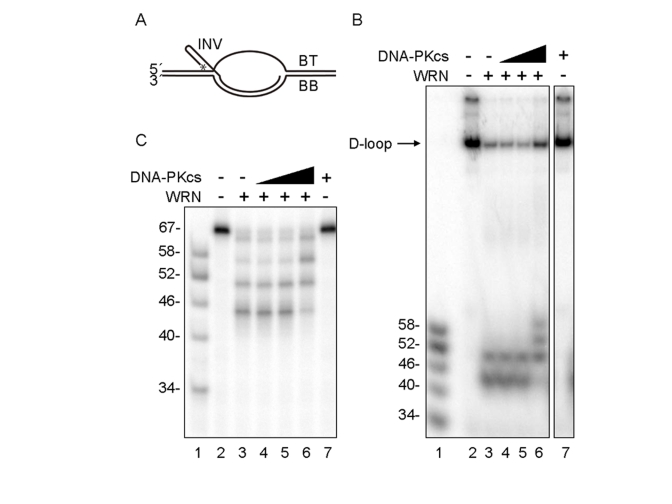
D-loop unwinding by WRN in the absence and presence of DNA-PKcs. (**A**) The D-loop substrate consisted with INV, BT and BB.  5'-end of INV was radiolabeled as indicated by asterisk.  WRN (3.3 nM, lanes 3-6) and increasing amounts of DNA-PKcs (0.67 nM, lane 4; 3.3 nM, lanes 5 and 7; 16.7 nM, lane 6) were incubated in standard reaction buffer prior to addition of the telomeric D-loop substrate.  Reaction products were analyzed by native (**B**) or denaturing gel electrophoresis (**C**). Lanes 1 in (**B**) and (**C**): A DNA ladder marker.

 Here, we report findings that add novel insight into the function of WRN and DNA-PKcs at telomeres.  DNA-PKcs stimulates WRN helicase activity on D-loop substrates. Measurements of telomere length revealed that G-tail shortenings in DNA-PKcs-deficient cells were reversed by overexpression of WRN helicase.  We propose that the DNA-PKcs and WRN cooperation play a critical and interactive role in maintaining telomere length and structure in proliferating cells.

## Results

### DNA-PKcs modulates WRN processing of telomeric D-loops

The effect of DNA-PKcs on WRN was analyzed using an *in vitro* telomeric D-loop unwinding assay.  The DNA substrate used in this assay consists of a bubble with two 30 bp duplex arms separated by a 33 nt ssDNA "melted" region, one strand of which is annealed to an "invading" ssDNA (INV) (Figure 1A).  The melted region and the invading ssDNA carry telomeric repeats, such that the DNA substrate mimics a telomeric D-loop.  Previous studies with this DNA substrate showed that WRN exonuclease partially degrades and the WRN helicase unwinds and releases the INV DNA strand, which is stable after release because WRN exonuclease does not efficiently degrade ssDNA [12].  In this study, the DNA substrate was incubated with WRN in the absence or the presence of increasing amounts of DNA-PKcs.  Under these conditions, WRN was not phosphorylated by the DNA-PKcs since Ku70 and Ku80 are absent.  Reaction products were analyzed by native and denaturing gel electrophoresis, as shown in Figures 1B and 1C, respectively.  In the absence of PKcs, WRN released 52- and 46-mer ssDNA products (Figure 1B and 1C, lanes 3), consistent with its pausing at the GGG sequence in the telomeric repeat, as reported previously [12].  In the presence of up to a 5-fold molar excess of DNA-PKcs to WRN, the ssDNA reaction products were longer, primarily 52-, 58-, and 64- nucleotides in length (Figures 1B and 1C, lanes 6).  However, the total ssDNA product (and the amount of unreacted DNA substrate) was similar in WRN reactions with or without DNA-PKcs (Figures 1B and 1C, lanes 6).  These results suggested two possibilities; i) the processivity of WRN exonuclease is inhibited by DNA-PKcs, or ii) the processivity of WRN helicase is stimulated by DNA-PKcs.

### DNA-PKcs stimulates WRN helicase activity on telomeric D-loops

**Figure 2.**
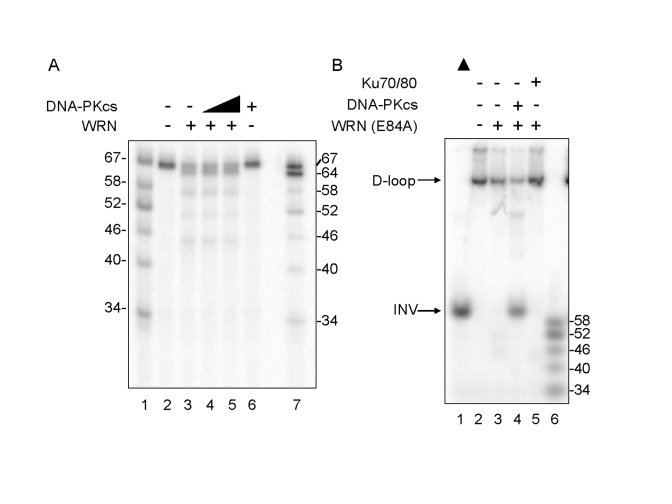
** Differential Effect of DNA-PKcs on WRN helicase and exonuclease activities.**  (**A**) WRN (3.3 nM, lanes 3-5) and DNA-PKcs (3.3 nM, lane 4; 16.7 nM, lanes 5 and 6) were incubated in standard reaction buffer lacking ATP prior to addition of the D-loop substrate.  Reaction products were analyzed by denaturing gel electro-phoresis.  Lanes 1 and 7: A DNA ladder marker.  (**B**) WRN (E84A) (3.3 nM, lanes 3-5) was preincubated with either DNA-PKcs (16.7 nM, lane 4) or Ku (3.3 nM, lane 5) in standard reaction buffer prior to addition of the D-loop substrate.  Reaction products were analyzed by native gel electrophoresis.  Lane 1: heat-denatured D-loop substrate denoted by a filled triangle.  Lane 6: A DNA ladder marker.

The effect of DNA-PKcs on WRN enzymatic functions was examined by incubating WRN with telomeric D-loop substrates in the absence of ATP, which inactivates WRN helicase without affecting WRN exonuclease.  Under these conditions, WRN exonuclease produced 64-, 58-, 52- and 46-mer reaction products, and the distribution of reaction products was unchanged by addition of DNA-PKcs (Figure 2A).  Thus, DNA-PKcs does not inhibit WRN exonuclease.  The ability of DNA-PKcs to stimulate WRN helicase activity was examined by incubating an exonuclease-deficient point mutant, WRN (E84A) with telomeric D-loop substrates in the absence or presence of DNA-PKcs.  WRN (E84A), which has a normal level of helicase activity but no exonuclease activity, unwinds 3.3% of the telomeric D-loop substrate in the absence of DNA-PKcs (Figure 2B, lane 3) and unwinds 66% of the substrate in the presence of DNA-PKcs, producing a full-length INV (Figure 2B, lane 4).  This very significant stimulation is not observed in reactions containing Ku 70/80 (Figure 2B, lane 5), arguing against the possibility that a low level contamination of DNA-PKcs with Ku is responsible for the observed stimulation of WRN helicase.  These results suggest that DNA-PKcs stimulates WRN helicase, possibly by increasing its processivity, and that this stimulation is independent of WRN exonuclease.

### DNA-PKcs does not stimulate BLM helicase activity on telomeric D-loops

**Figure 3.**
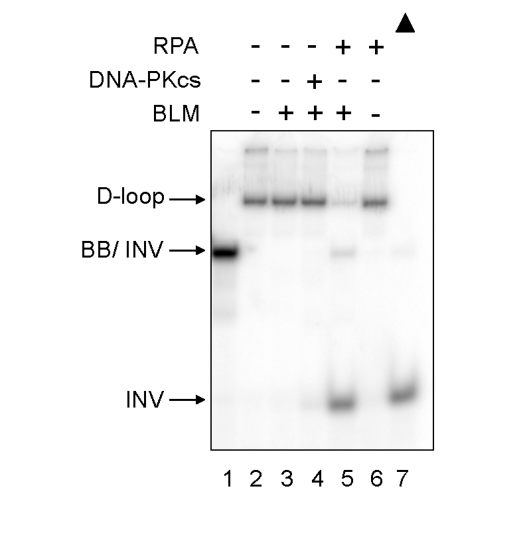
** Differential effect of DNA-PKcs on WRN and BLM helicase activities.**  BLM (3.3 nM, lanes 3-5) and either DNA-PKcs (16.7 nM, lane 4) or RPA (16.7 nM, lanes 5 and 6) were incubated in standard reaction buffer prior to addition of the D-loop substrate.  Lane 1: A DNA marker, [^32^P]-INV annealed with BB.  Lane 7: heat-denatured D-loop substrate denoted by a filled triangle.

BLM is a human RecQ family helicase, which like WRN, is proposed to play a role at telomeres in human cells [14]. Therefore, the effect of DNA-PKcs on BLM ability to unwind telomeric D-loop DNA substrates was examined (Figure 3).  In reactions containing a low concentration of BLM, BLM failed to unwind the telomeric D-loop in the absence or presence of DNA-PKcs.  However, when replication protein A (RPA) was added to the same amount of BLM, BLM helicase fully unwound the telomeric D-loop, producing full-length INV, as previously reported [13].  Thus, DNA-PKcs does not stimulate BLM helicase, indicating that its interaction with WRN helicase is specific.

### DNA-PKcs stimulates WRN helicase activity on non- telomeric D-loops

**Figure 4.**
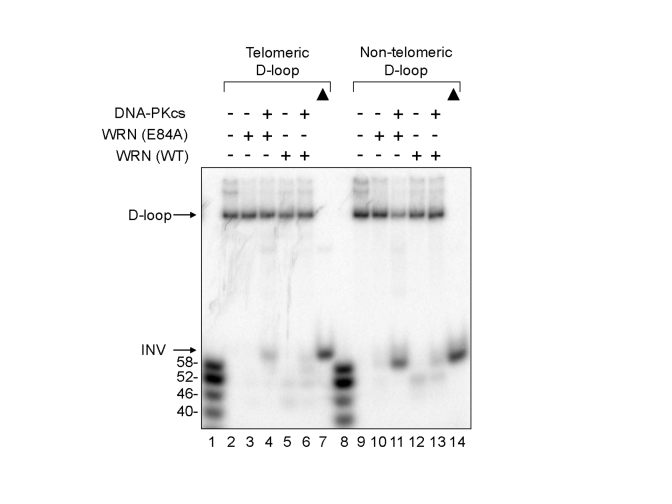
** Effect of DNA-PKcs on WRN helicase activity on telomeric and non-telomeric D-loops.**  WRN wild type (WT) (3.3 nM, lanes 5, 6, 12, and 13) or WRN (E84A) (3.3 nM, lanes 3, 4, 10, and 11) was preincubated with DNA-PKcs (16.7 nM, lanes 4, 6, 11, and 13). A telomeric (lanes 2-6) or a non-telomeric D-loop substrate (lanes 9-13) was added to the reaction.  Lanes 1 and 8: A DNA ladder marker.  Lanes 7 and 14: heat-denatured telomeric and non-telomeric D-loop substrates, respectively, denoted by filled triangles.

The ability of DNA-PKcs to stimulate WRN wild type or WRN (E84A) helicase on non-telomeric D-loop substrates was also examined (Figure 4).  The results show that wild type and WRN (E84A) unwinds a small fraction of the non-telomeric D-loop substrate in the absence of DNA-PKcs, and the addition of DNA-PKcs increased the unwinding, while it enabled WRN to produce longer ssDNA products (Figure 4, lanes 9-13).  Similar results were observed with telomeric D-loop DNA substrates, as observed in Figures 1B and 2B (Figure 4, lanes 2-6).  These results suggest that DNA-PKcs may stimulate WRN helicase activity on D-loop structures in telomeric or non-telomeric DNA because the stimulation appears to be independent of the nucleotide sequence of the DNA substrate *in vitro*.

### DNA-PKcs does not stimulate WRN helicase on non-D-loop DNA substrates

**Figure 5.**
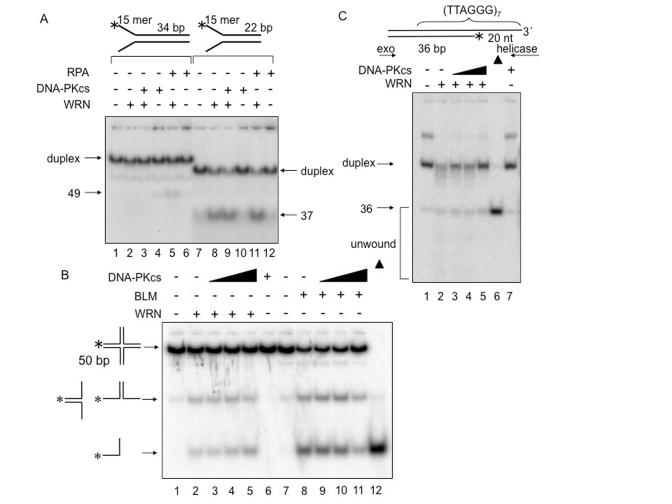
** DNA-PKcs fails to alter WRN helicase activity on forked duplex, Holliday junction and G-tailed telomeric DNA substrates.**  DNA helicase assays were carried out in the presence of the indicated proteins and DNA substrates.  (**A**) WRN (1 nM, lanes 2, 3, 5, 8, 9, and 11) and either DNA-PKcs (5 nM, lanes 3, 4, 9, and 10) or RPA (5 nM, lanes 5, 6, 11, and 12) were incubated in standard reaction buffer prior to addition of a 34 bp forked duplex (0.5 nM, lanes 1-6) or a 22 bp forked duplex (0.5 nM, lanes 7-12).  (**B**) WRN (4 nM, lanes 2-5) or BLM (2.5 nM, lanes 8-11), and DNA-PKcs (4 nM, lane 3; 8 nM, lane 4; 20 nM, lanes 5; 2.5 nM, lane 9; 5 nM, lane 10; 12.5 nM, lane 11) were incubated with in HJ reaction buffer prior to addition of Holliday junction (0.5 nM, lanes 1-11). Lane 6: DNA-PKcs (20 nM) alone. Lane 12: heat-denatured Holliday junction denoted with filled triangles.  (**C**) G-tailed duplex (0.5 nM, lanes 1-5 and 7) was incubated with WRN (7.5 nM, lane 2-5) and DNA-PKcs (6.25 nM, lane 3; 12.5 nM, lane 4; 25 nM, lanes 5 and 7) in standard reaction buffer.  Lane 6: heat-denatured G-tailed duplex denoted by a filled triangle.

The ability of DNA-PKcs to stimulate WRN helicase was also tested on several DNA metabolic intermediates other than D-loops (Figures 5).  These included two forked duplexes with poly-T 15-mer arms, one with a 34-bp duplex region containing (TTAGGG)_4_ and one with a 22-bp duplex region lacking telomeric repeats (Figure 5A).  WRN helicase unwinds the 34-bp forked duplex in the presence of RPA (Figure 5A, lane 5), as reported previously [23].  Under the same conditions but in the presence of DNA-PKcs, WRN did not unwind this DNA substrate (Figure 5, lane 3).  WRN unwinds the 22-bp forked duplex with similar efficiency in the absence or presence of DNA-PKcs or RPA (Figure 5A, lanes 8, 9 and 11).  Although RPA is thought to increase the processivity of WRN helicase, the intrinsic processivity of WRN helicase appears to be sufficient for unwinding the 22-bp forked duplex used in this experiment.  Figure 5B shows that WRN and BLM helicase unwind a Holliday junction DNA substrate, and that this activity is not stimulated by DNA-PKcs.  These results indicate that DNA-PKcs stimulates the processivity of WRN helicase on the D-loop substrate but not on other DNA substrates examined in this study.  Because D-loops may be enriched in telomeric regions *in vivo*, this is consistent with the proposed roles of WRN and DNA-PK specifically in telomere length maintenance.

Telomeric DNA can exist in a closed D-loop form or an open form, with the open form more likely to occur during DNA replication or in response to DNA damage.  Therefore,the ability of DNA-PKcs to stimulate WRN helicase was also tested on a telomeric DNA substrate that resembles the telomere in an open conformation (Figure 5C).  For this purpose, a DNA substrate was prepared containing a telomericduplex DNA upstream of G-tail [24].  Note that the polarity of WRN helicase is 3'-5', allowing it to unwind duplexes with a G-tail, but not duplexes with a 5'-ssDNA tail.  The DNA substrate used in these experiments includes both a G-tailed duplex as depicted in Figure 5C and a second species, which is likely to be a bi-molecular G-quadruplexstructure formed by annealing of the ssDNA tails of two G-tailed duplexes.  The latter structure has a slower electrophoretic mobility than the G-tailed duplex (Figure 5C, lane 1),and it is destabilized by WRN (Figure 5C, lanes 2-5) or boiling(Figure 5C, lane 6).

WRN exonuclease degrades the open telomeric DNA substrate starting at the3'-OH blunt end, and WRN helicase unwinds and releases the shortened strand from theG-tailed duplex (Figure 5C, lane 2).  DNA-PKcs did not stimulate WRN helicase on this DNA substrate (Figure 5C, lanes 2-5).  The results suggest that DNA-PKcs stimulates WRN helicase on a telomeric D-loop substrate, but not on a G-tailed DNA duplex, an open form of a telomeric D-loop.

### Protection of G- tail by WRN helicase activity

**Figure 6.**
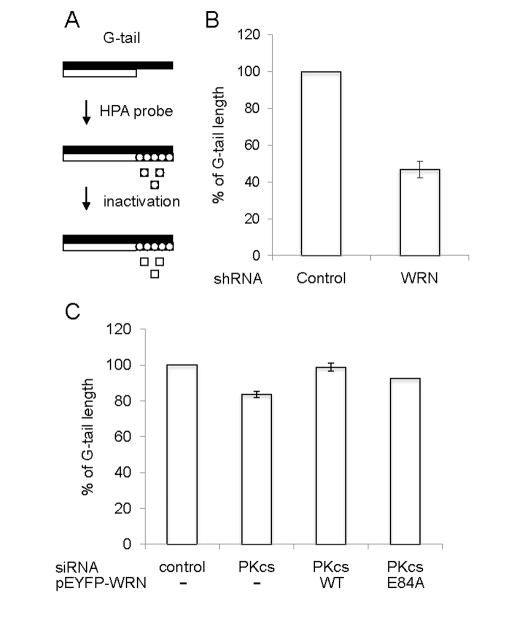
** Quantification of telomere G-tail length by hybridization protection assay in DNA-PKcs knockdown U-2 OS cells.**  (**A**) A schematic of the HPA for telomere G-tail.  Non-denatured genomic DNA was incubated with acridinium ester (AE)-labeled 29-mer telomere HPA probe. The AE of unhybridized and mis-hybridized probes was hydrolyzed, and chemilumines-cence from AE of hybridized probes was measured.  (**B** and **C**) G-tail length of cells expressing an shRNA control or an shRNA against WRN was examined in panel B.  G-tail length of cells transfected with siRNA against control (left), siRNA against DNA-PKcs (middle left), siRNA against DNA-PKcs with pEYFP-WRN (middle right), or siRNA against DNA-PKcs with pEYFP-WRN (E84A) (right) was examined in panel **C**.  The G-tail length in the control cells was represented as 100%.  Data are represented as mean +/- standard errors of two independent experiments.

The above studies suggest that DNA-PKcs stimulates telomere unwinding by WRN *in vitro*, but do not address whether this interaction is important *in vivo*.  Nevertheless, previous studies are consistent with this possibility.  In particular, telomere length decreases more quickly in Terc^-/-^/DNA-PKcs^-/-^ mice than in Terc^-/-^ mice [25], and Terc^-/-^/WRN^-/-^ but not WRN^-/-^ mice have a telomere dysfunction [26].  Thus, experiments were performed to test whether the interaction between WRN and DNA-PKcs is important for telomere length maintenance *in vivo* (Figure 6).  For this purpose, a G-tail telomere hybridization protection assay (HPA) was performed with DNA purified from U-2 OS cells, which are telomerase negative.  The G-tail telomere HPA assay, shown schematically in Figure 6A, accurately measures telomere G-tail length.  The G-tail telomere HPA assay was first performed using U-2 OS cells which stably express an shRNA targeted to WRN or control U-2 OS cells which stably express a scrambled shRNA with no significanthomology to known human genes (Figure 6B).  The results show that G-tail length is significantly shorter in WRN knockdown cells.  The effect of DNA-PKcs on the G-tail length was examined using the cells transfected with an siRNA targeted to DNA-PKcs (Figure 6C).  G-tail length was also slightly shorter in DNA-PKcs knockdown cells, compared to cells transfected with control siRNA.  Overexpression of N-terminally EYFP-tagged WRN (E84A), an exonuclease dead mutant, in the DNA-PKcs knockdown cells reversed the G-tail shortening.  This suggests that endogenous WRN exonuclease is responsible for a part of this outcome of the shortening in the absence of DNA-PKcs, and an excess amount of WRN (E84A) prevents the exonuclease from attacking the G-tail and exhibit unwinding activity.  However, a similar result was obtained by overexpression of N-terminally EYFP-tagged WRN wild type in the DNA-PKcs knock down cells.  There may be a mechanism to support an access of an exonuclease domain of WRN (E84A) but not WRN wild type to the G-tail in cells (Figure 6C), because the domain (1-239 amino acids) is important to regulate its binding to forked duplex, which is resemble a part of D-loop substrate [27].

## Discussion

**Figure 7.**
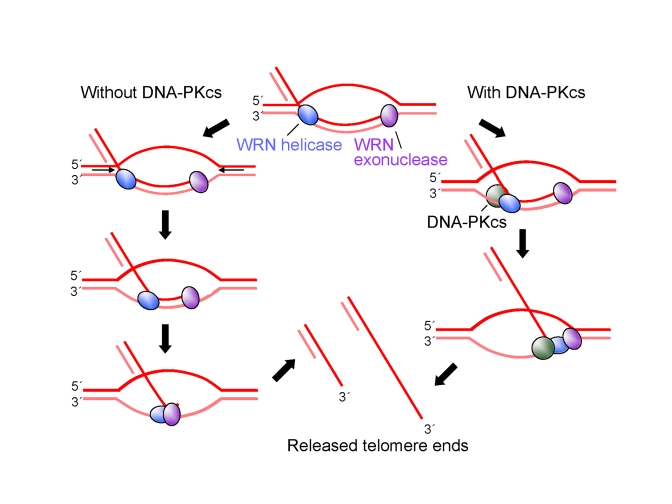
** A model for protection of G-tails by DNA-PKcs.**  See text for detailed description of the model.

This study demonstrates that DNA-PKcs stimulates the apparent processivity of WRN helicase but not WRN exonuclease on telomeric and non-telomeric D-loop substrates *in vitro* and that overexpression of WRN helicase reverses telomere G-tail shortening *in vivo* caused by knockdown of DNA-PKcs in U-2 OS cells.  Based on these results, we propose a model for the role of WRN and DNA-PKcs in D-loop unwinding (Figure 7).  The key points of the model are as follows: 1) In the absence of DNA-PKcs and WRN exonuclease, WRN helicase dissociates from DNA prior to release of a full-length invading strand, resulting in reannealing of the unwound region; 2) when WRN exonuclease degrades the 3' tail of the invading strand, WRN helicase releases the shortened invading strand, even in the absence of DNA-PKcs; 3) DNA-PKcs stimulates WRN processivity, so that exonuclease-deficient WRN or WRN is able to release an intact or nearly intact invading strand from the D-loop, respectively.  This mechanism would protect telomeric DNA 3'-ends, prevent telomere shortening, and potentially avoid p53-p21-dependentreplicative senescence.

 The results also indicate that DNA-PKcs stimulates WRN-catalyzed unwinding of non-telomeric D-loop, implying that WRN and DNA-PKcs could cooperate to unwind recombination-associated D-loops in genomic regions other than the telomere.  This is consistent with a possible role of WRN in processing D-loop intermediates in homologous recombination, which is supported by several *in vitro* studies [8,28].

 Previous studies also show that POT1 and RPA, WRN and BLM interacting proteins, stimulate WRN and BLM-catalyzed unwinding of telomeric D-loop substrates *in vitro *[13].  However, the mechanism(s) of this stimulation may differ from the mechanism by which DNA-PKcs stimulates WRN helicase.  POT1 and RPA are ssDNA binding proteins. They stabilize ssDNA and prevent ssDNA reannealing, rather than preventing WRN dissociation from the substrate through their interaction with WRN.  Unlike POT1 and RPA, DNA-PKcs has a low affinity for ssDNA, but high affinity for junctions between ssDNA and dsDNA [29].  Thus, DNA-PKcs might bind to the ssDNA/dsDNA junctions of D-loops that have been partially melted by WRN and prevent ssDNA reannealing.  Direct interaction between WRN and DNA-PKcs was demonstrated [21].  It is also possible that DNA-PKcs prevents WRN from dissociating from the DNA substrate, and that this interaction stimulates the processivity of WRN helicase.  Recently, it was reported that deacetylation of histone H3 lysine 9 by  SIRT6 is required for the stable association of both WRN and DNA-PKcs with telomeric chromatin, suggesting the possibility of a role for SIRT6 to control the interaction between WRN and DNA-PKcs at telomeres [30,31]. Additional studies are needed to determine whether and how Ku70/80 influences the interaction between DNA-PKcs and WRN, especially because Ku70/80 binds tightly to WRN and stimulates its exonuclease activity [32].

 Mouse model studies indicate that both DNA-PKcs deficiency and WRN deficiency synergize with telomerase loss and shortened telomeres to accelerate the onset of aging related phenotypes.  Mice deficient in both WRN and telomerase recapitulate most of the premature aging WS phenotypes in the later generation cohorts that experienced telomere shortening [26,33]. Similarly, mice rendered doubly deficient in DNA-PKcs and telomerase exhibited accelerated aging-related degenerative phenotypes including tissue atrophy, compared to singly null mice, and this was further exacerbated in later generations [34].  The loss of either WRN or DNA-PKcs in telomerase deficient mice was associated with accelerated telomere shortening and chromosome fusions [26,34]. Preservation of the telomeric tail is essential for preventing telomere dysfunction and chromosome fusions [35], and our biochemical data suggest that WRN and DNA-PKcs cooperate to prevent telomere tail shortening during processing at telomeric ends in telomerase deficient cells.

## Methods

Cells. U-2 OS cells stably transfected with a vector expressing an shRNA plasmid against control or WRN were grown in monolayer culture in Dulbecco's Modified Eagle Medium (DMEM) (Invitrogen) supplemented with 10% fetal bovine serum, 0.2 mg/ml hygromycin B (Invitrogen), 50 μg/mlstreptomycin, and 50 U/ml penicillin as previously reported [36].  The U-2 OS cells stably expressing untargeted shRNA was transfected with siRNA against DNA-PKcs (sc-35200, Santa Cruz Biotechnology) or control siRNA (sc-37007, Santa Cruz Biotechnology), with or without pEYFP-WRN or pEYFP-WRN (E84A) vector, using lipofectamine 2000 Reagent (Invitrogen).  Cells were incubated for 2 days and harvested.

Plasmids. DNA fragment encoding wild type WRN and WRN (E84A) was excised from pBK-WRN and pBK-WRN (E84A) with *Sal*I and *Ssp*I, and ligated into the *Sal*I and *Sma*I site of pEYFP-C1 vector (Clontech) to produce pEYFP-WRN and pEYFP-WRN (E84A) vectors, respectively.

Proteins. His-tagged WRN wild type (WT), WRN (E84A) and human Ku 70/86 were overexpressed in and purified from Sf9 insect cells using a baculovirus expression system as previously described [37,38].  Recombinant human RPA was purified from *E. coli *BL21(DE3) pLysS transformed with p11d-tRPA as described previously [39].  DNA-PKcs was purified from placenta as described previously [40].  His-tagged BLM was prepared from yeast strain JEL-1 [41].

G-tail telomere HPA assay. The G-tail length was quantified by the HPA using genomic DNA as described previously[44].  Signal intensity for each sample was normalized by DNA concentration using NANO drop.

DNA substrate. BB, BT and 5'-radiolabeled INV were annealed to form a telomeric D-loop [12].  BBmx, BT and 5'-radiolabeled INVmx were annealed to form a non-telomeric D-loop [13]. 5'-Radiolabeled and unlabeled 37-mer oligonucleotides were annealed to form a 22-bp forked duplex [42], an oligonucleotide 6 and a 5'-radiorabeled oligonucleotide 5 were annealed to form a 34-bp forked duplex [23], and X12-2, X12-3, X12-4 and 5'-radiolabeled X12-1 were annealed to form a Holliday junction [43].  The G-tailed substrate consisting of a 36-bp duplex with 14-bp of unique sequence followed by 22-bp of (TTAGGG)_3_TTAG sequence and a 20 nt 3'-ssDNA tail of the sequence GG(TTAGGG)_3_ was constructed as previously described [24].  Briefly the substrate was formed by annealing the 5'-end labeled Tel Tail duplex oligonucleotide [24] into a hairpin to promote correct alignment of the telomeric repeats.  An annealing reaction was at 95˚C for 5 min, cooled stepwise (1.2 C˚/min) to 60˚C, incubated for 1 hr, and then cooled stepwise (1.2 C˚/min) to 25˚C.  The hairpin was digested with *Eco*RV (New England BioLabs) to generate a blunt end, and the substrate was purified by PAGE.

Helicase and exonuclease assays. D-loop unwinding reactions were performed as described previously [12].  Briefly, the indicated amount of WRN or BLM was preincubated with DNA-PKcs, RPA or Ku on ice prior to addition of DNA.  Assays contained 0.5 nM [^32^P] 5' end-labeled D-loop substrate in 30 μl of standard reaction buffer [40 mM Tris-HCl (pH 8.0), 4 mM MgCl_2_, 5 mM DTT, 0.1 mg/ml BSA and 2 mM ATP].  Aliquots of 20 and 5 μl were electrophoresed in non-denaturing 8% polyacrylamide gels containing 0.1% SDS and 14% denaturing gels, respectively.  Reaction products were quantified using a PhosphorImager and ImageQuantsoftware (Molecular Dynamics).  Reactions using forked duplex and G-tailed telomere substrates were performed in 20 μl standard reaction buffer [23,24].  Reactions using Holliday junction substrates were performed in 20 μl HJ reaction buffer [40 mM Tris-HCl (pH 8.0), 2 mM MgCl_2_, 5 mM DTT, 0.1 mg/ml BSA and 2 mM ATP] [43].
